# Effects of spatial resolution and noise on gamma analysis for IMRT QA

**DOI:** 10.1120/jacmp.v15i4.4690

**Published:** 2014-07-08

**Authors:** Jessie Y. Huang, Kiley B. Pulliam, Elizabeth M. McKenzie, David S. Followill, Stephen F. Kry

**Affiliations:** ^1^ Department of Radiation Physics The University of Texas M.D. Anderson Cancer Center Houston TX USA

**Keywords:** gamma index, IMRT QA, measurement noise, measurement resolution

## Abstract

We investigated the sensitivity of the gamma index to two factors: the spatial resolution and the noise level in the measured dose distribution. We also examined how the choice of reference distribution and analysis software affect the sensitivity of gamma analysis to these two factors for quality assurance (QA) of intensity‐modulated radiation therapy (IMRT) treatment plans. For ten clinical IMRT plans, the dose delivered to a transverse dose plane was measured with EDR2 radiographic film. To evaluate the effects of spatial resolution, each irradiated film was digitized using three different resolutions (71, 142, and 285 dpi). To evaluate the effects of image noise, 1% and 2% local Gaussian noise was added to the film images. Gamma analysis was performed using 2%/2 mm and 3%/3 mm acceptance criteria and two commercial software packages, OmniPro I'mRT and DoseLab Pro. Dose comparisons were performed with the treatment planning system (TPS)‐calculated dose as the reference, and then repeated with the film as the reference to evaluate how the choice of reference distribution affects the results of dose comparisons. When the TPS‐calculated dose was designated as the reference distribution, the percentage of pixels with passing gamma values increased with both increasing resolution and noise. For 3%/3 mm acceptance criteria, increasing the film image resolution by a factor of two and by a factor of four caused a median increase of 0.9% and 2.6%, respectively, in the percentage of pixels passing. Increasing the noise level in the film image resulted in a median increase in percentage of pixels passing of 5.5% for 1% added local Gaussian noise and 5.8% for 2% added noise. In contrast, when the film was designated as the reference distribution, the percentage of pixels passing decreased with increased film noise, while increased resolution had no significant effect on passing rates. Furthermore, the sensitivity of gamma analysis to noise and resolution differed between OmniPro I'mRT and DoseLab Pro, with DoseLab Pro being less sensitive to the effects of noise and resolution. Noise and high scanning resolution can artificially increase the percentage of pixels with passing gamma values in IMRT QA. Thus, these factors, if not properly taken into account, can potentially affect the results of IMRT QA by causing a plan that should be classified as failing to be falsely classified as passing. In designing IMRT QA protocols, it is important to be aware that gamma analysis is sensitive to these parameters.

PACS number: 87.55.Qr, 87.55.km, 87.56.Fc

## I. INTRODUCTION

The gamma (γ) index introduced by Low et al.^(1)^ is a quantitative method of comparing two dose distributions and is routinely used for quality assurance (QA) of intensity‐modulated radiation therapy (IMRT) treatments. Typically, a two‐dimensional measured dose distribution is compared with the planar dose calculated by the treatment planning system (TPS). Performing dose comparisons using the gamma index involves the choice of the dose difference criterion, the distance to agreement (DTA) criterion, and the designation of the reference distribution (either the measured or calculated dose distribution). For each point in the reference distribution, the gamma index is calculated by comparing this point to all points in the evaluated dose distribution within a given search radius, and the gamma index is calculated based on the point in the evaluated distribution that best satisfies both the dose difference and DTA criterion. A gamma value ≤1 indicates that the point has passed the acceptance criteria, whereas a gamma value >1 indicates a failing point. Typically, the percentage of points that have passing gamma values determines the overall results of IMRT QA (i.e., whether a particular treatment plan has passed or failed). For instance, a common acceptance criterion is that at least 90% of points need to pass 3%/3 mm criteria for a plan to be considered passing.^2^, ^3^

Despite its routine use for IMRT QA, gamma index calculations are potentially sensitive to a number of factors not commonly taken into account. For instance, Low and Dempsey^(4)^ noted that calculation of the gamma index can be affected by the presence of noise in dose distributions. This makes sense if one considers a single point in the reference distribution with a dose of 104 cGy and a uniform 100 cGy evaluated dose distribution. For a 3%/3 mm criteria, this point would fail, since there are no points in the evaluated distribution that satisfy the 3% dose difference criterion. However, if the evaluated distribution includes noise (as all measurements would), say at a 1% random noise level (such that the standard deviation of dose values is 1 cGy), then approximately 16% of the points would now fall within the 3% dose difference criterion, simply based on the normal distribution. This random noise “provide(s) opportunities to locate a point in the evaluated distribution that (is) closer than the DTA that would be determined with no noise.”^(4)^ Therefore, a higher noise level in the evaluated distribution will result in a higher probability of calculating a passing gamma value. Noise and statistical uncertainty are inherent to measurement devices used for IMRT QA.^(5)^ For film dosimetry, the noise level and the signal‐to‐noise ratio are dependent on the number of photons detected by the film,^(6)^ as well as nonuniformities in the film composition, variations in the temperature and chemical concentrations in the film processor, and noise introduced in the digitization of the film.^7^, ^8^, ^9^, ^10^, ^11^ Under optimal conditions, the dose uncertainty can be less than 3% for EDR2 film, but this uncertainty is not commonly accounted for in the gamma index calculation.^(12)^ Although this study focuses on film, other 2D measurement devices commonly used for IMRT QA, such as diodes and ion chamber arrays, also have measurement uncertainty.^(13)^ Noise and uncertainty are also inherent to dose distributions generated using Monte Carlo simulations, in which the statistical uncertainty is governed by computation time.^(14)^ Although Low and Dempsey^(4)^ explored the effect of noise on the gamma index using simple, proof‐of‐principle square‐field test cases, the effect of noise on patient‐specific IMRT QA scenarios remains unexplored in the literature.

The spatial resolution of both the evaluated and reference distributions can also affect the results of IMRT dose comparisons.^15^, ^16^ In this study, a higher scan “resolution” refers to film that is digitized to have smaller pixel sizes and thus a higher density of data points in the film image. An evaluated distribution with a higher spatial resolution than its corresponding reference distribution will have a large number of points within a given search radius for each point in the reference distribution. This large number of comparison points offers greater opportunity to find a point that satisfies the acceptance criteria. It is therefore possible that, for the same comparison, an evaluated distribution at a lower resolution (i.e., fewer points) could result in a failing gamma value, while the comparison with the evaluated distribution sampled at a higher resolution (i.e., more points) could pass, especially if more noise is introduced as a result of sampling at a finer resolution. Low and Dempsey^(4)^ recommend that the spatial resolution of the evaluated distribution be at least one‐third of the DTA criterion, but make no recommendations regarding the maximum spatial resolution for comparisons for which there is a resolution disparity (e.g., higher resolution film vs. lower resolution TPS dose grid). Bailey et al.^(17)^ investigated the effects of detector resolution on passing rates by comparing a high density detector (large number of sample points) against a lower density detector (fewer sample points). They found that the passing rates obtained with the lower density detector exhibited some variation due to statistical uncertainty, following a normal distribution centered on the result obtained with the high density detector.

Another important, and often overlooked, consideration is the clinical effect of the assignment of evaluated and reference distributions for the gamma index calculation. The recommendation by Low and Dempsey^(4)^ is that the measured distribution be assigned as the reference and the calculated distribution be assigned as the evaluated distribution. However, in practice, there is not a standard assignment for the reference distribution, and many IMRT QA software packages allow the user to input either distribution as the reference or evaluated distribution. At the University of Texas MD Anderson Cancer Center, it is standard to assign the reference distribution opposite to the Low and Dempsey recommendation. However, these assignments are not trivial, since the calculation of the gamma index is not symmetric with respect to which distribution is used as the reference.^(4)^ For example, gamma values are underestimated (i.e., agreement is overestimated) if there is noise in the evaluated distribution, while noise in the reference distribution adds noise to the gamma index distribution.^(4)^

Due to a lack of studies evaluating the clinical effect of noise, resolution, and assignment of reference distribution on the gamma index for routine IMRT QA, we evaluated the effect of these parameters for ten patient‐specific IMRT QA treatment plans.

## II. MATERIALS AND METHODS

### A. Film irradiation

Ten clinical IMRT plans representing a variety of treatment sites (four head and neck, one genitourinary, two gynecologic, two gastrointestinal, and one mesothelioma) were chosen for this study. Six of these plans passed our institution's IMRT patient‐specific QA based on film and ion chamber measurements, while four of them failed one or both QA metrics. The IMRT QA hybrid plans were created in the Pinnacle^3^ TPS (Philips Healthcare, Fitchburg, WI) on the CT dataset of a water‐equivalent I'mRT Body Phantom (IBA Dosimetry, Schwarzenbruck, Germany), and the planar dose was calculated using a 1 mm×1 mm×1 mm dose grid. Each plan was delivered on a Clinac 21EX accelerator (Varian Medical Systems, Palo Alto, CA) with a single Kodak Extended Dose Range 2 (EDR2) Ready Pack Film (Eastman Kodak, Rochester, NY) inserted into the transverse plane of the phantom. That is, IMRT QA was performed using a composite delivery with all beams delivered at the planned gantry angles. In addition, a calibration film was irradiated using eight dose levels ranging from 77 to 587 cGy to generate a calibration curve for conversion of optical density to dose. Films were developed using a Kodak RP X‐OMAT Processor (Eastman Kodak) and were digitized using the VXR‐16 Dosimetry Pro or Dosimetry Pro Advantage (VIDAR Systems Corporation, Herndon, VA). The VIDAR scanner uses a fluorescent white light source with a spectral distribution between 250 and 750 nm coupled to a linear CCD system to measure light transmission. The scanner allows the choice of three scan resolutions (71, 142, or 285 dpi) and produces a 16‐bit depth grayscale image.^18^, ^19^ The detector elements have a resolution of 570 dpi. Lower scan resolutions are achieved by averaging the signal from multiple detector elements. Image registration between the film and TPS planar dose was performed using pinpricks in the film at known locations. Relative dose comparisons were performed by normalizing both dose distributions. Comparisons were performed using a 10% dose threshold, two different acceptance criteria (2%/2 mm and 3%/3 mm), and global normalization (i.e., dose difference criterion is with respect to the normalization dose, typically the maximum dose in the dose plane). These parameters were chosen based on common gamma criteria and dose thresholding used clinically.^(2)^

### B. Noise, resolution, and interplay between the two

To evaluate the effect of noise, various levels of local Gaussian noise (1% and 2% standard deviation) were added to 71 dpi digital film images. The percentage of pixels passing obtained with these noisy images was then compared with that of the original 71 dpi image with no added noise.

To investigate how film image resolution affects gamma comparison results, each of the irradiated films was digitized at three different resolutions (71, 142, and 285 dpi) and gamma comparisons were performed for each film image. The percentage of pixels passing for the higher resolution film images (142 and 285 dpi) was then compared with that of the 71 dpi film image.

To investigate whether digitizing the film at a higher resolution resulted in more noise in the digital image, we also scanned our calibration film using different resolutions. We then quantified the noise level at each of the eight dose levels by measuring the standard deviation of the pixel values using a 0.20 cm^2^ region of interest.

### C. IMRT QA software

Gamma analysis was performed using two different commercial IMRT QA software packages, OmniPro I'mRT (IBA Dosimetry) and DoseLab Pro (Mobius Medical Systems, LP, Houston, TX). These two software packages differ slightly in their implementation of dose comparisons using the gamma index. For example, DoseLab Pro automatically uses a bilinear interpolation algorithm to downsample the higher resolution film image to match the resolution of the TPS dose grid. OmniPro I'mRT also has the ability to change the resolution of either the film or TPS dose distributions. However, no resizing was performed for comparisons using OMNIPro I'mRT. Furthermore, OmniPro I'mRT and DoseLab Pro perform dose normalization and image registration slightly differently, which can affect the direct comparison of results between these software packages. Therefore, our study was not intended to compare absolute results between the different software packages, but rather to identify trends associated with image noise and resolution within each individual software package's implementation of the gamma calculation, and to evaluate the level of sensitivity of each to these factors.

### D. Choice of reference distribution

OmniPro I'mRT allows the choice of which dose distribution to set as the reference. Therefore, with this software, comparisons were made with the TPS dose distribution as the reference and repeated with the film dose distribution as the reference. Conversely, the DoseLab Pro software does not allow the user to designate a distribution as the reference. Rather, the measured dose distribution is always the reference distribution, and the TPS calculation is always the evaluated distribution.^(20)^ Therefore, only comparisons with the film as the reference distribution were performed using DoseLab Pro.

### E. Statistical analysis

In order to determine if any observed changes in passing rates due to noise or resolution were statistically significant, two‐sided paired Student's *t*‐tests were performed for each variable studied. Furthermore, linear regression analysis was performed to see if the change in passing rate was dependent on the baseline passing rate (i.e., the passing rate obtained without added noise at a resolution of 71 dpi). An F‐test for simple linear regression was performed to determine if the there was a significant linear relationship (95% confidence level).

## III. RESULTS

### A. Noise

**Figure 1:**
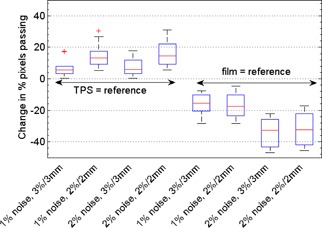
Box plots depicting how IMRT QA results obtained using the OMNIPro I'mRT software depend on noise level for our ten treatment plans. Shown is the change in percentage pixels passing caused by various levels of added Gaussian noise (1% and 2%), compared with the original 71 dpi film image with no added noise. Comparisons with various acceptance criteria (2%/2 mm and 3%/3 mm) and choice of reference distribution (TPS=reference and film=reference) are shown.

Figure 1 shows the effect of added noise on the percentage of pixels passing, as determined with use of OMNIPro I'mRT. The change in the percentage of pixels passing that is reported is the difference in the percentage of pixels passing for the noisy film image and the original (no added noise) film image (Eq. (1)):

(1) $$change\;in\;\%pixels\;passing={\left(\%\;pixels\;passing\right)}_{noisy}-{\left(\%pixels\;passing\right)}_{noaddednoise}$$

**Figure 2:**
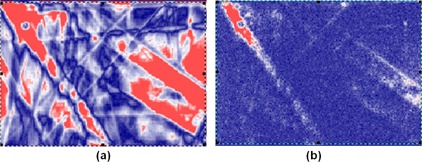
Gamma index maps showing the effect of increased image noise when the film is designated as the evaluated dose distribution: (a) shows the resulting gamma index map for the original 71 dpi film image, and (b) shows the gamma index map after 1% local Gaussian noise is added to the film image. Blue indicates a passing point, whereas red indicates a failing point.

For comparisons with the TPS as the reference, the percentage of pixels passing increased for increased noise in the film image. For 1% added local Gaussian noise, the median increase in percentage of pixels passing was 5.5% for 3%/3 mm and 13.1% for 2%/2 mm. For 2% added noise, the corresponding values were 5.8% for 3%/3 mm and 14.5% for 2%/2 mm. It should be noted that for most of these comparisons, the percentage of passing pixels reached 99%–100% with just 1% noise added. The effects of added noise may have been even more remarkable had the percentage of passing pixels started at a lower value for these plans or if a more sensitive metric (absolute gamma values) had been used to quantify the effects of image noise. Notably, the treatment plans that originally failed our institution's IMRT QA were dramatically affected by the addition of noise. The outliers shown in Fig. 1 are from failing plans. For one of these failing plans, the percentage of pixels passing increased by 17.5% with the addition of 1% noise for 3%/3 mm acceptance criteria. Figure 2 illustrates the effect of added noise on the gamma index distribution for one of our failing plans.

For the reverse comparisons (film=reference) in OMNIPro I'mRT, the opposite trend was observed. The percentage of pixels with passing gamma values decreased with increased film noise (Fig. 1). For 1% added noise, the median change in percentage of pixels passing was −15.5% for 3%/3 mm and −17.4% for 2%/2 mm. For 2% added noise, the corresponding values were −32.5% for 3%/3 mm and −32.4% for 2%/2 mm.

**Figure 3:**
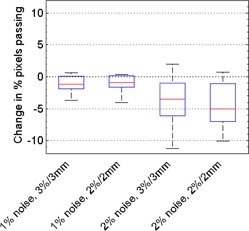
Box plots depicting how IMRT QA results obtained using DoseLab Pro depend on noise level for our ten treatment plans. Shown is the change in percentage pixels passing caused by various levels of added Gaussian noise (1% and 2%), compared with the original 71 dpi film image with no added noise. Comparisons with various acceptance criteria (2%/2 mm and 3%/3 mm) are shown. All gamma comparisons using DoseLab were performed with the film as the reference distribution.

Figure 3 shows the effect of image noise on gamma comparison results using DoseLab Pro. Film was the reference for these comparisons, and the percentage of pixels passing generally decreased with increased film noise (p<0.05). For our ten treatment plans, the median change in percentage of pixels passing for 1% added noise was −1.2% for 3%/3 mm and −0.9%

2%/2 mm. For 2% added noise, the corresponding values were −3.5% for 3%/3 mm and −5.1% for 2%/2 mm. Compared with the OmniPro I'mRT results, the percentage of pixels passing in DoseLab Pro was less affected by the addition of film noise. The results of our noise study for both OmniPro I'mRT and DoseLab Pro are summarized in Table 1.

**Figure 4:**
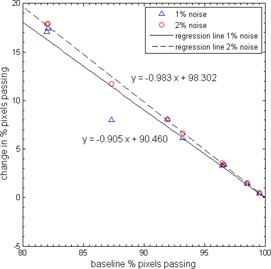
Plot showing the relationship between baseline passing rate and change in passing rate due to added noise for comparisons performed with OMNIPro I'mRT (3%/3 mm,TPS=reference). The slopes shown are statistically significant (p<0.05 for F‐test for simple linear regression).

To investigate whether the change in passing rate was dependent on the baseline passing rate (obtained with no added noise), linear regression analysis was performed. It was found that there was a significant linear relationship (p<0.05) when the TPS was the reference in OmniPro I'mRT, indicating that a lower baseline passing rate (e.g., failing plan) will have a larger increase in passing rate when noise is introduced to the film image (Fig. 4). No notable or significant trends were observed for DoseLab Pro comparisons or OmniPro I'mRT comparisons with the film as the reference distribution.

**Table 1:** Summary of statistically significant changes in percentage of pixels passing due to changes in film resolution and noise for dose comparisons performed with 3%/3 mm mm gamma criteria. P‐values are shown in parenthesis

	*OMNIPro I'mRT*		*DoseLab Pro*
	TPS=reference	Film=reference	(Film=reference)
x2 resolution	↑(0.01)	↔	↔
x4 resolution	↑(0.01)	↔	↔
1% noise	↑↑(0.01)	↓↓↓(<0.001)	↓(0.03)
2% noise	↑↑(0.01)	↓↓↓(<0.001)	↓(0.02)

One arrow=a mean change in percentage pixels passing of<5%; two arrows=≥5% and <10%; three arrows=a change≥10%; horizontal arrow=no statistically significant change in percentage pixels passing.

### B. Resolution

**Figure 5:**
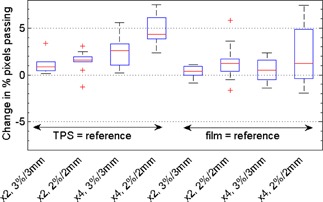
Box plots depicting how IMRT QA results obtained using the OMNIPro I'mRT software depend on the scan resolution of the film image for our ten treatment plans. Shown is the change in percentage pixels passing caused by changes in film resolution. “x2” indicates an increase in resolution from 71 to 142 dpi, and “x4” indicates an increase from 71 to 285 dpi. Comparisons with various acceptance criteria (2%/2 mm and 3%/3 mm) and choice of reference distribution (TPS=reference and film=reference) are shown.

The effect of increased film scan resolution on the percentage of pixels with passing gamma values is shown in Fig. 5 for the OmniPro I'mRT software. When the TPS‐calculated dose distribution was designated as the reference, the percentage of pixels passing increased with increasing film resolution. Specifically, in going from a film resolution of 71 to 142 dpi, the median increase in percentage of pixels passing was 0.9% for 3%/3 mm and 1.6% for 2%/2 mm acceptance criteria. Increasing the scan resolution by a factor of 4 to 285 dpi resulted in a median increase of 2.6% for 3%/3 mm and 4.3% for 2%/2 mm. When film was the reference, there was no consistent trend observed (i.e., some plans showed an increase in passing rates, while some showed a decrease). Overall, there was no statistically significant change in passing rates for increased scan resolution when the film was the reference distribution (p>0.05).

**Figure 6:**
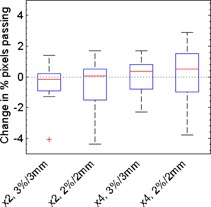
Box plots depicting how IMRT QA results obtained using the DoseLab Pro software depend on the scan resolution of the film image for our ten treatment plans. Shown is the change in percentage pixels passing caused by changes in film resolution. “x2” indicates an increase in resolution from 71 to 142 dpi, and “x4” indicates an increase from 71 to 285 dpi. Comparisons with various acceptance criteria (2%/2 mm and 3%/3 mm) are shown. All gamma comparisons using Doselab Pro were performed with the film as the reference distribution.

The results for dose comparisons using DoseLab Pro, for which only comparisons where the film was the reference were performed, are shown in Fig. 6. DoseLab Pro showed little dependence on the resolution of the film image, as evidenced by all the medians in Fig. 6 being close to zero. The largest median change in percentage of pixels passing was only 0.5% for a four‐fold increase in resolution at 2%/2 mm. Overall, there was no statistically significant change in passing rates for different film scan resolutions using the DoseLab Pro software (p>0.05). However, as with the OmniPro software, individual plans could show sizeable variations in percent of pixels passing. The results of our resolution study are summarized in Table 1.

Based on our linear regression analysis, there was no significant relationship between baseline passing rates and change in passing rates due to higher resolution scanning for either OmniPro I'mRT or DoseLab Pro comparisons.

### C. Interplay between resolution and noise

**Figure 7:**
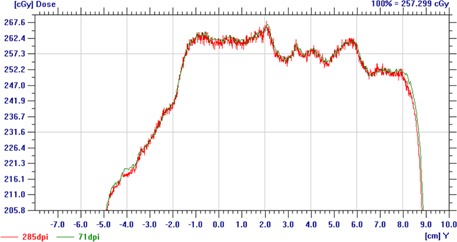
A dose profile from OmniPro I'mRT showing the increased noise resulting from digitizing film at higher scan resolutions (285 vs. 71 dpi).

By digitizing our calibration film at various scan resolutions, we found that at a given dose level, the percent standard deviation was greater for films digitized at a higher resolution. For instance, the percent standard deviation at a dose of 77 cGy was 0.7% when the film was digitized at a resolution of 71 dpi and increased to 1.3% at 285 dpi. For a dose level of 2.2 Gy, the standard deviation increased from 0.7% at 71 dpi to 0.8% at 285 dpi. The fact that digitizing films at a higher resolution can cause film images to contain more noise can be seen in Fig. 7, which shows dose profiles obtained for the same piece of irradiated film digitized at 71 and 285 dpi. More noise can be seen in the dose profile from the 285 dpi film image.

## IV. DISCUSSION

Our study on the effect of film noise and resolution on dose comparisons using the gamma metric demonstrated that these parameters can artificially increase (or decrease) the percentage pixels passing, possibly masking (or exaggerating) the dosimetric differences that gamma analysis is designed to highlight. Additionally, we showed the clinical importance of the assignment of reference and evaluated distributions, which is not arbitrary. Rather, this choice determines whether the agreement between two dose distributions becomes better or worse with increased noise and higher spatial resolution. Finally, we showed that the sensitivity to noise and resolution of various IMRT QA software packages can vary greatly and, therefore, an understanding of how the software handles these parameters is essential for proper IMRT QA protocol design. Table 1 summarizes the trends found in this study.

Our study of noise using OmniPro I'mRT (Fig. 1) showed that image noise in the film caused an increase in the percentage of pixels with passing gamma values when the film was the evaluated distribution. This trend is consistent with the finding by Low and Dempsey^(4)^ that noise in the evaluated distribution increases the passing rate because it “provide(s) opportunities to locate a point in the evaluated distribution that (is) closer than the DTA that would be determined with no noise.” This effect was especially large for our failing plans. Introduction of just 1% random noise in one of these failing plans increased the percentage of pixels passing by 17.5% for 3%/3 mm criteria. This particular plan should be identified as failing (82% pixels passing at 3%/3 mm), but instead, the result of the added noise was to inflate the percentage of pixels passing such that the plan would be falsely identified as passing the acceptance criteria (99% pixels passing at 3%/3 mm). To put this 1% noise into perspective, we observed approximately 1% local noise in our calibration films for the dose levels typically used in IMRT QA, in agreement with previous studies for EDR2 film.^12^, ^21^ Therefore, noise at the level of 1% is relevant for this type of dosimeter. We also found that for these comparisons, there was a significant linear relationship between baseline passing rate (obtained when no additional noise was added) and the increase in percentage of pixels passing after noise was added for OMNIPro I'mRT comparisons where the TPS was the reference. That is, the lower the baseline passing rate, the more the percentage of pixels passing is increased due to noise. This is particularly troubling because the plans with lower passing rates (i.e., the plans that should be failing) are the most susceptible to the effects of noise.

It should be noted that we used global normalization in this study, meaning that the 3% dose difference criteria is with respect to a global normalization dose value (typically the maximum dose), following our clinical practice. However, the noise added to film images in this study was local, not global, Gaussian noise. Gamma analysis performed using local normalization is generally a more sensitive metric, and the ability of noise to inflate passing rates may have been more drastic had local normalization been used in the calculation of the gamma index.^(22)^

When the same dose comparisons were performed with the noisy film as the reference distribution, the opposite trend was observed and the percentage of pixels passing decreased with increased noise. Therefore, the presence of noise can have a dramatic effect on the results of dose comparisons using the gamma metric, and the amount of noise in the measured dose distribution should be minimized as much as possible.

In the resolution portion of this study, we found that the results of gamma analysis were dependent on the number of measurements points obtained, or rather the resolution of the measured data, in agreement with the study performed by Bailey et al.^(17)^ For the OmniPro I'mRT software, we found that increased resolution caused the percentage of pixels passing to increase when the TPS was the reference, while resolution had little effect on passing rates when the film was the reference distribution. The largest change was observed when the spatial resolution of the film image was increased by a factor of four, resulting in a median increase of percentage of pixels passing of 4.3%.

Furthermore, we also found that noise and resolution are interrelated, as digitizing film at an increased resolution was associated with increased noise in the film image. For the VIDAR scanner, the lower scan resolutions are achieved by averaging the signal from multiple CCD detectors. Therefore, more noise, likely both statistical and electronic, is present in the high‐resolution images in comparison to the lower resolution images. This added noise associated with high‐resolution film digitization further increases the probability of finding a passing point, leading to an increase in gamma passing rates, although a smaller magnitude increase than those observed in the noise portion of this study. Therefore, the additional noise associated with increased resolution is most likely of smaller magnitude than the artificial noise introduced to the film images in the noise portion of this study.

With the film designated as the reference, there was little dependence of gamma results on film resolution (median change in percentage of pixels passing <2%) using OmniPro I'mRT. Therefore, the effects of spatial resolution on IMRT QA results can be minimized by designating the higher resolution dose distribution as the reference, choosing to digitize at a resolution more comparable to the TPS dose grid, or choosing software that appropriately handles the effects of unequal resolution.

Compared with OmniPro I'mRT, DoseLab Pro was less sensitive to the effects of image noise and resolution. For instance, the addition of 1% random noise to the film image resulted in a median change in the percentage of pixels passing of −15.5% with OmniPro I'mRT and only −1.2% with DoseLab Pro (3%/3 mm,film=referencee). Similarly, in OnmiPro I'mRT, increasing the resolution of the film affected the percentage of pixels passing, whereas for DoseLab Pro, the change was negligible (a median change of <1%). DoseLab Pro's relative insensitivity to these two factors can be explained by the fact that this software automatically uses interpolation to downsample the higher resolution film image to have the same resolution as the TPS dose grid. It is important to note that different IMRT QA software packages have different implementations of the gamma index calculation, and these subtleties can affect the results of dose comparisons. Therefore, understanding how the software implements gamma analysis is an important step in choosing the appropriate IMRT QA acceptance criteria, especially for software that performs postprocessing of raw measurement data. The variability in passing rates obtained using different software packages is in general concerning. This variability could perhaps be minimized, or at least quantified, by using “standard test datasets” as commissioning tests for IMRT QA software.

Although our study focused on dose comparisons using film, these results also bear consideration for other devices commonly used for IMRT QA. All measurement devices are subject to some level of noise, so it is important to be aware of the level of noise associated with an IMRT QA device and to understand how that noise can affect the results of gamma analysis. With respect to resolution, our study is limited in that the results are most directly applicable to high‐density detectors used for IMRT QA, such as film and EPID, and less applicable to lower density detectors, such as fixed arrays of diodes or ion chambers. For these lower density detectors, the measured dose distribution is usually at a coarser resolution than the TPS dose plane.^23^, ^24^ This disparity in resolution is analogous to the disparity between the TPS and film, except now TPS resolution is higher than the measured resolution. For these array detectors, the trends observed in this study may not apply or may be heavily dependent on the software package used and whether the TPS is designated as the reference.^25^, ^26^ Though outside the scope of the current study, investigation of how noise and resolution affect these commercial lower density devices is warranted.

## V. CONCLUSIONS

Our results have shown that the percentage of pixels passing in gamma analysis for IMRT QA is sensitive to both scan resolution and the presence of noise in the film image. Sensitivity to these factors was dependent on choice of software, as well as on choice of reference distribution. The presence of noise in the film image had a drastic effect on the results of gamma analysis, inflating the percentage of passing points to such a degree that failing plans could be falsely identified as passing. Film resolution had a lesser effect on the results of gamma analysis. Designating the measured (film) distribution as the reference appears to make the comparison less sensitive (more robust) to the effects of resolution, although there is still some impact. It is necessary to have a good understanding of these factors (i.e., image noise, image resolution, choice of reference, and choice of software) in order to thoughtfully design IMRT QA protocols and guidelines such that delivery errors are not masked by factors that can underestimate gamma values and artificially inflate the number of passing points.

## ACKNOWLEDGMENTS

This work was supported by Public Health Service grants CA 10953 and CA 081647, awarded by the National Cancer Institute, Department of Health and Human Services. We would like to thank Luke Whittlesey, Miguel Herrera, and Nicholas Murray for their help with film measurements, as well as Tamara Locke for her assistance in editing this manuscript.
